# The Value of Warning Signs From the WHO 2009 Dengue Classification in Detecting Severe Dengue in Children

**DOI:** 10.1097/INF.0000000000004326

**Published:** 2024-04-19

**Authors:** Mulya Rahma Karyanti, Cuno S. P. M. Uiterwaal, Sri Rezeki Hadinegoro, Indah Suci Widyahening, Siti Rizny F. Saldi, J. A. P. Hans Heesterbeek, Arno W. Hoes, Patricia Bruijning-Verhagen

**Affiliations:** * From the Department of Child Health, Faculty of Medicine Universitas Indonesia, Jakarta, Indonesia; † Julius Center for Health Sciences and Primary Care, University Medical Center Utrecht and Utrecht University, Utrecht, Netherlands; ‡ Department of Community Medicine, Faculty of Medicine Universitas Indonesia, Jakarta, Indonesia; § Clinical Epidemiology and Evidence-Based Medicine Unit, Cipto Mangunkusumo Hospital, Faculty of Medicine Universitas Indonesia, Jakarta, Indonesia; ¶ Department of Population Health Sciences, Faculty of Veterinary Medicine, Utrecht University, Utrecht, Netherlands.

**Keywords:** warning signs, WHO 2009, dengue, severe dengue, cohort study, children

## Abstract

**Background:**

World Health Organization proposed 7 warning signs to identify the risk of severe dengue in 2009. This study aimed to evaluate the value of these warning signs in detecting severe dengue in children.

**Material and Methods:**

A cross-sectional study was conducted utilizing data of children with clinical dengue infection obtained from medical records between January 2009 and December 2018 in Jakarta. Children with confirmed dengue were analyzed and stratified into 3 age groups: infants less than 1 year old, children 1–14 years and adolescents 15–18 years of age. Positive predictive value, negative predictive value (NPV), sensitivity and specificity of each warning sign present or absent on admission in detecting severe dengue were computed.

**Results:**

Six hundred ninety-nine children with clinical dengue infection were enrolled, among whom 614 (87.8%) had confirmed dengue infection, either by antigen or antibody serological tests. Severe dengue occurred in 211/614 (34.4%) cases. In infants, important warning signs on admission to detect or exclude severe dengue were liver enlargement (NPV 80.8%) and clinical fluid accumulation (NPV 75%). In children and adolescents, warning sign with highest NPV (in children 76.6% and in adolescents 91.9%) was increase in hematocrit concurrent with a rapid decrease in platelet count. Other warning signs with high NPV values in children were abdominal pain (72%), vomiting (70%), clinical fluid accumulation (69.3%), and in adolescents’ abdominal pain (80.7%), vomiting (75.7%), clinical fluid accumulation (82.7%). NPVs increase with more than 1 warning sign in all age groups.

**Conclusion:**

In infants, liver enlargement or clinical fluid accumulation are important warning signs for severe dengue, when both are absent, severe dengue is unlikely. In older children and adolescents, an increase in hematocrit with the concurrent rapid decrease in platelet count is most discriminative; followed by the absence of abdominal pain, vomiting or fluid accumulation are unlikely severe dengue.

Dengue virus infection has a wide spectrum, with manifestations ranging from very mild to severe, sometimes lethal forms. Severe dengue can lead to intravascular leakage and, without adequate supportive therapy, multiple organ failure and even death.^1^ The World Health Organization (WHO) reported that about 3.9 billion people living in 128 countries are at risk of contracting dengue. Annually, an estimated 50–100 million cases of dengue infection occur worldwide, and the number of deaths increased from 16,957 in 1990 to 40,467 in 2017.^2,3^ This makes dengue an important public health problem globally.^4^ According to the 1997 WHO Dengue Classification (WHO-1997), symptomatic dengue virus infections are grouped into 3 categories: undifferentiated fever, dengue fever and dengue hemorrhagic fever (DHF). The DHF cases are further classified into 4 severity grades, with grades III and IV being defined as dengue shock syndrome (DSS).^5^ Changes in the epidemiology of dengue led to criticism on the usefulness and applicability of this classification in clinical care and in 2009 the WHO issued a new guideline that classifies clinical dengue as: (1) dengue without warning signs, (2) dengue with warning signs and (3) severe dengue (WHO-2009).^6^

The new guideline includes warning signs (WSs) that help early identification of (imminent) DSS, severe bleeding manifestations or severe organ impairment.^7^ Delay in diagnosis, referral to healthcare and fluid management during the critical phase in dengue disease progression can lead to higher mortality in severe dengue cases, and therefore early detection of disease progression is crucial. The WSs included in the guideline were selected to support healthcare professionals in resource-limited healthcare settings in the clinical assessment of dengue-infected patients.^8^ The selection was based on usability tests conducted in clinical settings across 18 countries^9^ and resulted in a more practical and acceptable guideline for clinicians, although not all dengue-endemic countries have applied the new guidelines to date.^9–11^

Timely recognition or ruling of severe dengue among children with dengue is notoriously difficult. Studies on the performance of WSs to predict severe dengue in pediatric care are rare, while WSs might also differ between infants and older children. The diagnostic value of WSs in different age groups thus needs to be explored in larger groups of dengue dengue-infected cases, of whom some develop severe dengue. Evidence on the value of WS should be given attention to prevent complications and mortality. The International Classification of Disease (ICD) 10th revision currently employs WHO-1997, but WHO has revised it in ICD 11, incorporating the diagnosis of dengue based on WHO-2009 criteria. This revision came into effect on January 1, 2022 (source: https://www.who.int/news-room/detail/18-06-2018-who-releases-new-international-classification-of-diseases-(icd-11).

In Indonesia, children with severe dengue need referral to a secondary or tertiary care hospital center and hence adequate discrimination of children with severe versus nonsevere dengue in primary healthcare facilities is essential. The aim of this study was to evaluate the diagnostic value of WHO-2009 WSs on admission to the hospital in detecting severe pediatric dengue, with patients stratified by age group into infants, children 1–14 years old and adolescents.

## MATERIAL AND METHODS

### Study Design and Population

This study was conducted in a tertiary care hospital, Cipto Mangunkusumo, Jakarta, Indonesia, by collecting data from medical records in a 10-year period from January 2009 until December 2018. The hospital uses standardized daily assessment sheets for all patients with a clinical diagnosis of dengue infection, including results of physical examination, vital signs, laboratory parameters and radiological examinations. Clinical data and laboratory findings were obtained daily until discharge. All children up to 18 years old diagnosed with clinical dengue infection based on the WHO-1997 dengue criteria were assessed in detail.

Case definition for dengue fever includes an acute febrile illness with 2 or more of the following manifestations: headache, retro-orbital pain, myalgia, arthralgia, rash, hemorrhagic manifestations, leukopenia and supportive serology (positive IgM antibody test). DHF is defined as having the following symptoms: acute fever for 2–7 days, biphasic, hemorrhagic tendencies, thrombocytopenia <100,000 cells per mm and "evidence of plasma leakage due to increased vascular permeability, manifested by at least one of the following: a rise of hematocrit equal to or greater than 20% above average for age, sex and population, a drop in hematocrit following volume-replacement treatment equal to greater than 20% of baseline, signs of pleural effusion, ascites and hypoproteinemia". Dengue shock syndrome is defined as the presence of all 4 of the above-mentioned DHF criteria with evidence of circulatory failure. From these, we selected children with laboratory-confirmed dengue infection for inclusion in our study. Patients with nondengue or proven hematologic disorders, malignancy or incomplete medical record data were excluded.

### Laboratory Confirmed Dengue Infection

Serological confirmation tests of nonstructural 1 (NS1) dengue antigen, or IgM, IgG dengue antibody were performed from acute blood samples. Dengue viral infection was confirmed by positive NS1 detection using Dengue NS1 Ag Strip (Panbio). Primary dengue infection was defined when only IgM was positive, secondary dengue infection was defined when both IgM and IgG were positive and indeterminate dengue (prior dengue infection) was defined when IgG was positive but IgM was negative. Nondengue was defined when both IgG and IgM were negative. For dengue serology, the presence of dengue IgM and IgG in acute-phase serum was assessed using a rapid immunochromatographic test (Panbio Dengue Duo Cassette).

### Outcome Definition

Patients were classified as having nonsevere or severe dengue according to WHO-2009 case definitions. Severe dengue includes: (1) severe plasma leakage with shock (DSS) or presence of hypotension, tachycardia and signs of poor capillary perfusion with or without narrow pulse pressure; (2) severe bleeding, including bleeding from the gastrointestinal tract with or without the need for transfusions of blood products or (3) severe organ impairment defined as elevated levels of aspartate transaminase or alanine transaminase of 1000 IU/L or higher, central nervous system impaired consciousness or heart and other organ involvement.^7^ An independent trained physician classified the clinical dengue cases to classify patient outcomes as nonsevere dengue or severe dengue.

### Definition of WSs on Admission

The WSs considered included: abdominal pain, persistent vomiting, clinical fluid accumulation, mucosal bleeding, lethargy, liver enlargement and laboratory results showing an increase in hematocrit concurrent with a rapid decrease in platelet count. Abdominal pain was defined as abdominal tenderness and continuous (ie, not intermittent) pain. Persistent vomiting was defined as more than 3 episodes of vomiting within 12 hours. Fluid accumulation was defined as pleural effusion visible on a chest radiograph or ultrasound and ascites detected by abdominal ultrasound. Mucosal bleeding was defined as bleeding gums or conjunctiva, epistaxis, vaginal bleeding, hemoptysis or hematuria. Lethargy was defined as an alteration of consciousness with a Glasgow score of less than 15. Hepatomegaly was defined based on the liver edge palpation of more than 2 cm below the right costal margin. Increased hematocrit concurrent with a rapid decrease in platelet count (high hematocrit/low platelets) was defined as any increase in hematocrit from baseline at admission in the febrile phase, with a concurrent rapid decrease in platelet count of at least 10,000 cells/mm^3^ in 24 hours or with a drop of platelet count below 100,000 cells/mm.^3,12^ The Presence or absence of each warning sign on admission was enumerated per patient.

### Statistical analysis

All laboratory-confirmed cases of dengue infection were included in the analysis. Patients were stratified into 3 age groups, namely infants (less than 1 year old), children (1–14 years old) and adolescents (15–18 years old). Clinical WSs were compared between patients with and without severe dengue using *χ*^2^ tests or Fisher exact tests where appropriate. We assessed the value of each warning sign to discriminate between severe and nonsevere dengue using the following diagnostic test criteria: positive predictive value, negative predictive value (NPV), sensitivity and specificity, stratified by age group. Next, we computed the diagnostic test criteria for combinations of WSs. Data were analyzed by using SPSS version 22 (IBM, Chicago) and MedCalc’s diagnostic test evaluation calculator (https://www.medcalc.org/calc/diagnostic_test.php).

## RESULTS

A total of 699 medical records were reviewed for children with clinical dengue admitted to Cipto Mangunkusumo Hospital during the study period. Of these, 614 (87.8%) cases with confirmed dengue infection were included in the study (figure, Supplemental Digital Content 1, http://links.lww.com/INF/F494). The 85 cases excluded consisted of 75 nondengue patients and 10 patients with incomplete medical records. Of the confirmed dengue cases, 139/614 (22.6%) were classified as primary dengue infection, 304/614 (49.5%) as secondary dengue infection, 159/614 (25.9%) as indeterminate (prior dengue infection) and 12 (2%) as positive NS1 dengue. Based on the WHO-2009 classification, 403 (65.6%) of the 614 confirmed dengue cases were classified as nonsevere dengue and 211 (34.4%) as severe dengue. The prevalence of severe dengue stratified by age group was 16/42 (38.1%) in infants, 181/506 (35.1%) in children and 14/63 (22.2%) in adolescents. The median age for children with nonsevere and severe dengue was 10 (IQR = 7) and 7 (IQR = 7) years, respectively. Baseline characteristics of confirmed dengue cases are shown in Table 1.

**TABLE 1.** Characteristics of Children with Confirmed Dengue Infection Admitted Between January 2009 and December 2018 (n = 614)

Characteristics	Non severe Dengue (%) N = 403 (65.6%)	Severe Dengue (%) N = 211 (34.4%)
Age group (years)		
<1	26 (6.4)	16 (7.5)
1–14	328 (81.3)	181 (85.7)
15–18	49 (12.1)	14 (6.6)
Gender		
Male	219 (54.3)	107 (50.7)
Female	184 (45.6)	104 (49.2)
Duration of fever (days)*	4 (1)	4 (1)
Length of stay (days)	4 (2)	4 (2)
Vital signs at admission <1 year*		
Systolic blood pressure	85 (12)	90 (17)
Diastolic blood pressure	58 (10)	52 (10)
Pulse rate	120 (25)	140 (47)
Respiration rate	28 (2)	30 (17)
Temperature on admission*	37 (1)	37 (0.9)
Vital signs at admission 1–14 years*		
Systolic blood pressure	100 (16.8)	100 (20)
Diastolic blood pressure	60 (10)	62 (10)
Pulse rate	100 (20)	110 (28.8)
Respiration rate	24 (5.3)	26 (10)
Temperature on admission*	37 (1.3)	36.8 (1.3)
Vital signs at admission 15–18 years*		
Systolic blood pressure	106 (10)	100 (8)
Diastolic blood pressure	70 (18)	70 (10.3)
Pulse rate	90 (16)	89.5 (26)
Respiration rate	20 (4)	24 (4.5)
Temperature on admission*	37 (1.5)	36.7 (1)
Hematologic findings at admission†		
Hemoglobin (g/dL)	13.2 ± 2.0	14.1 ± 2.6
Hematocrit (%)	39.3 ± 5.8	41.5 ± 7.5
Leucocyte (/µL)	4378.8 ± 3315.3	5957.5 ± 3834.6
Platelet (/µL)	88494.7 ± 46364.1	65477.3 ± 45971.3
Outcome		
Alive	403 (100)	205 (97.1)
Died	0 (0)	6 (2.8)

* Median (IQR).

† Mean ± standard deviation.

IQR indicates interquartile range.

**FIGURE 1.**
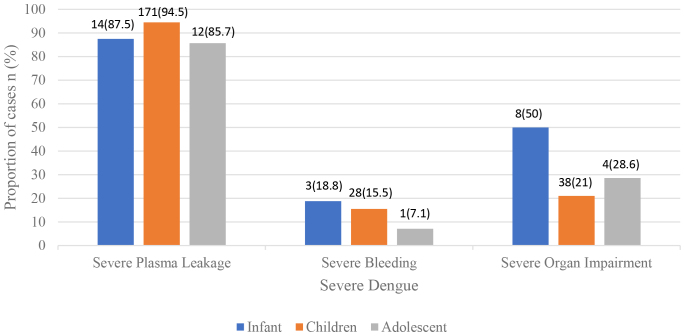
Prevalence of different manifestations of severe dengue by age group (n = 614).

**FIGURE 2.**
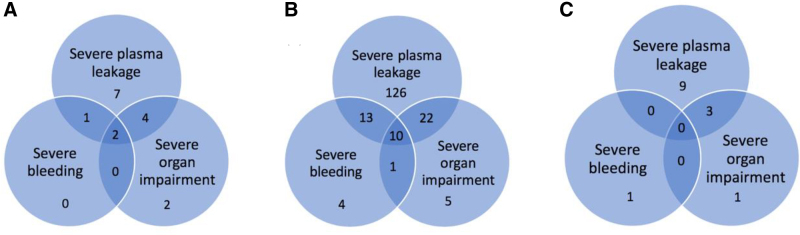
A: Infants with severe dengue (n = 16). B: Children with severe dengue (n = 181). C: Adolescents with severe dengue (n = 14).

The most common manifestation of severe dengue was severe plasma leakage in all age groups (Fig. 1). Severe bleeding and severe organ involvement occurred more frequently in infants compared to other age groups. Some patients had more than 1 manifestation of severe dengue, as depicted in Figure 2. Severe organ impairment in infants manifested as impaired consciousness, in 8 of 16 infant cases with severe dengue, whereas in children 1–14 years of age, it was mostly increased transaminase levels (>1000 IU/L, 7 cases) and cardiac involvement (2 cases). In adolescents, severe organ impairment manifested as increased transaminase levels in 2 cases, and as impaired consciousness in 2 cases. All 6 deaths occurred in the age group 1–14 years: 2 cases were 3 years, 2 were 5 years and 1 case each were 7 and 11 years of age.

WSs present at admission and stratified by age group are described in Table 2. Among the infants with severe dengue, the most common WSs were liver enlargement 11/16 (68.6%) and clinical fluid accumulation 10/16 (62.5%). In children 1–14 years with severe dengue, the 3 most common WSs were increase in hematocrit with rapid decrease of platelet count 122/181 (61.9%), abdominal pain 108/181 (59.7%) and persistent vomiting 95/181 (30.4%). In adolescents with severe dengue, the most common warning sign was increase in hematocrit with rapid decrease of platelet count 11/14 (78.6%).

**TABLE 2.** Warning Signs on Admission Among 614 Confirmed Dengue Patients Stratified by Age and by Severe or Nonsevere Dengue

Warning Signs	Total Dengue Cases n (%)	Nonsevere n (%)	Severe n (%)	*P* Value	PPV (%)	NPV (%)	Sn (%)	Sp (%)
Age <1-year patients (infants)	42 (100)	26 (61.9)	16 (38.1)					
Individual WS								
Abdominal pain or tenderness	4 (9.5)	2 (7.7)	2 (12.5)	0.628	50	63.1	12.5	92.3
Persistent vomiting	15 (35.7)	10 (38.5)	5 (31.3)	0.636	33.3	59.2	31.3	61.5
Clinical fluid accumulation	18 (42.9)	8 (30.8)	10 (62.5)	0.044	55.6	75	62.5	69.2
Mucosal bleeding	2 (4.8)	2 (7.7)	0 (0)	0.517	50	63.2	12.5	92.3
Lethargy, restlessness	4 (9.5)	2 (7.7)	2 (12.5)	0.628	50	63.2	12.5	92.3
Liver enlargement >2 cm	16 (38.1)	5 (19.2)	11 (68.8)	0.001	68.8	80.8	68.8	80.8
IHigh HCT/low platelets	13 (31)	8 (30.8)	5 (31.3)	0.974	38.5	62.1	31.3	69.2
Age 1–14 years (children)	509 (100)	328 (64.4)	181 (35.6)					
Individual WS								
Abdominal pain or tenderness	248 (48.7)	140 (42.7)	108 (59.7)	0.003	43.6	72.0	59.7	57.3
Persistent vomiting	222 (43.6)	127 (38.7)	95 (52.5)	0.003	42.8	70.0	52.5	61.3
Clinical fluid accumulation	98 (19.3)	43 (13.1)	55 (30.4)	0.000	56.1	69.3	30.4	86.9
Mucosal bleeding	80 (15.7)	54 (16.5)	26 (14.4)	0.533	49.1	68.0	28.8	83.5
Lethargy, restlessness	16 (3.1)	11 (3.4)	5 (3.1)	0.714	31.3	64.3	2.8	96.7
Liver enlargement >2 cm	120 (23.6)	61 (18.6)	59 (32.6)	0.000	49.2	68.6	32.6	81.4
High HCT/low platelets	214 (42)	102 (31.1)	122 (61.9)	0.000	52.3	76.6	61.9	68.9
Age 15–18 years (adolescents)	63 (100)	49 (77.8)	14 (22.2)					
Individual WS								
Abdominal pain or tenderness	32 (50.8)	24 (49)	8 (35.7)	0.590	25	80.7	57.1	51.0
Persistent vomiting	26 ((41.3)	21 (42.9)	5 (35.7)	0.632	19.2	75.7	35.7	57.1
Clinical fluid accumulation	11 (17.5)	6 (12.2)	5 (35.7)	0.041	45.4	82.7	35.7	87.8
Mucosal bleeding	5 (7.9)	4 (8.2)	1 (7.1)	1.000	20	77.6	7.1	91.8
Lethargy, restlessness	2 (3.2)	1 (2)	1 (7.1)	0.398	50	78.7	7.1	98
Liver enlargement > 2 cm	10 (15.9)	9 (18.4)	1 (7.1)	0.434	10	75.5	7.1	81.6
High HCT/low platelets	26 (41.3)	15 (30.6)	11 (78.6)	0.001	42.3	91.9	78.6	69.4

HCT, hematocrit; NPV, negative predictive value; PPV, positive predictive value; Sn, sensitivity; Sp, specificity.

In infants, liver enlargement was most discriminative with an NPV of 80.8 %, followed by clinical fluid accumulation (NPV: 75%). In older children, all WSs, except for mucosal bleeding and lethargy. Restlessness was significantly more common among patients with severe dengue. NPV was highest for an increase in hematocrit concurrent with a rapid decrease in platelet count (76.1 %). Other WSs with good NPVs in children 1–14 years were abdominal pain, persistent vomiting and clinical fluid accumulation. In adolescents, results on the discriminative value of individual WS were largely in line with results for 1–14-year-olds.

In infants, the combination of the 2 most discriminative WSs (liver enlargement and clinical fluid accumulation), had a NPV of 88%. In adolescents, several combinations of WS each had comparable discriminative power (Table, Supplemental Digital Content 2, http://links.lww.com/INF/F495), including abdominal pain and increase in hematocrit concurrent with rapid decrease in platelet count (NPV: 100%), increase in hematocrit concurrent with rapid decrease in platelet count and clinical fluid accumulation (NPV: 100%). When the number of WSs increases, the discriminatory ability, notably NPV and specificity, improves, especially in infants (Table, Supplemental Digital Content 3, http://links.lww.com/INF/F496).

## DISCUSSION

This study showed that in infants, the most discriminative warning sign for severe dengue is liver enlargement with positive predictive value of 68.8% and NPV of 80.8 %, followed by clinical fluid accumulation with NPV of 75%. In older age groups, an increase in hematocrit concurrent with a rapid decrease in platelet count was most discriminative WS with NPV (76.1% and 91.9% in children 1–14 years and adolescents, respectively). Other WSs with high NPVs in children 1–14 years and adolescents were abdominal pain, persistent vomiting and clinical fluid accumulation. In the absence of one of these 3 WSs in older children, it leads to unlikely-to-severe dengue.

The prevalence of severe dengue in our pediatric cohort was about 34.4%, which was higher compared to another study from Medan, Indonesia, where the severe dengue prevalence was 25% among children less than 18 years old,^13^ but lower than the estimate from another study among pediatric patients, by Pothapregada et al.^14^ in India, where severe dengue occurred in 40.6%. The high prevalence obtained in the present study is probably due to the fact that the subjects came from a national tertiary referral hospital, predominantly attracting severe pediatric dengue cases. Our result was also much higher than studies that included all ages.^15–17^

Clinical awareness and assessment of the WSs as proposed in the WHO-2009 guidelines^7^ is crucial to monitor the critical phase in dengue-infected patients. When specific warning signs with a high NPV are absent, this can be used to guide clinical decision-making on watchful waiting versus referral, as the post-test probability of severe dengue is substantially reduced in these subjects. While the specificity of the WS can also be a helpful measure, its impact on post-test probability may be low if the particular WS is uncommon in both diseased and undiseased (ie, the absence of the WS does not rule out disease). The NPV is, therefore, the most useful test characteristic to use in this context of clinical decision-making.

In infants WSs based on clinical assessment such as mucosal bleeding and lethargy each have an NPV of 63.2%, whereas when we combine 2 WSs of lethargy and liver enlargement, the NPV increases to 76.5%. WSs based on laboratory results of increased hematocrit and decreased platelet count, and those based on radiology examination to detect clinical fluid accumulation, have high NPVs, but these WSs can only be detected in healthcare facilities equipped with laboratory or radiology facilities. While new diagnostic tools such as NS1 serotype-specific IgG measured by ELISA or PCR-based techniques with rapid results to detect dengue virus are being developed to assist the assessment of disease severity, medical management still heavily relies on clinical judgment as the time from onset of WSs to severe illness in most dengue cases is typically less than 1 day and patients may progress toward hypovolemic shock and even death if adequate fluid therapy is not administered immediately.^18^ In addition, access to more advanced diagnostic testing and imaging may be limited in many primary healthcare settings, further stressing the importance of the clinical WSs in early patient assessment to reduce morbidity and mortality in children.^19^

The fact that the dengue cases in this study were from a tertiary hospital is a potential limitation as circumstances and prevalence in primary or secondary healthcare settings may differ, with consequences for the applicability of our conclusions. The strength of this study was that the patients were obtained from a 10-year period with complete datasets of clinical manifestations, laboratory and radiology findings monitored daily until discharge and focus on pediatrics with age stratification.

## CONCLUSIONS

In infants, important WSs to detect severe dengue are liver enlargement and clinical fluid accumulation; when both these WSs are absent in infants, severe dengue is unlikely. In older children, the presence of any of the WSs—an increase in hematocrit concurrent with a rapid decrease in platelet count, abdominal pain, vomiting or fluid accumulation—is likely severe dengue.

## Supplementary Material



## References

[1] Simmons Cameron JF Chau N Wills B. Dengue. N Engl J Med. 2012;366:1423–1432. 22494122 10.1056/NEJMra1110265

[2] Zeng Z Zhan J Chen L. Global, regional, and national dengue burden from 1990 to 2017: a systematic analysis based on the global burden of disease study 2017. EClinicalMedicine. 2021;32:100712. 33681736 10.1016/j.eclinm.2020.100712 PMC7910667

[3] Ferreira G. Global dengue epidemiology trends. Rev Inst Med Trop Sao Paulo. 2012;54:S5–S6. 23011450 10.1590/s0036-46652012000700003

[4] Bhatt S Gething PW Brady OJ. The global distribution and burden of dengue. Nature. 2013;496:504–507. 23563266 10.1038/nature12060 PMC3651993

[5] World Health Organization. Dengue haemorrhagic fever: diagnosis, treatment, prevention and control. 2nd ed. Geneva: WHO; 1997.

[6] Srikiatkhachorn A Rothman AL Gibbons RV. Dengue--how best to classify it. Clin Infect Dis. 2011;53:563–567. 21832264 10.1093/cid/cir451 PMC3202316

[7] World Health Organization. Guidelines fo diagnosis, treatment, prevention and control. 1st ed. Geneva: WHO; 2009. 23762963

[8] Hadinegoro SR. The revised WHO dengue case classification: does the system need to be modified?. Paediatr Int Child Health. 2012;32:33–38. 22668448 10.1179/2046904712Z.00000000052 PMC3381438

[9] Barniol J Gaczkowski R Barbato EV. Usefulness and applicability of the revised dengue case classification by disease: multi-centre study in 18 countries. BMC Infect Dis. 2011;11:106. 21510901 10.1186/1471-2334-11-106 PMC3098176

[10] Wakimoto MD Camacho LA Guaraldo L. Dengue in children: a systematic review of clinical and laboratory factors associated with severity. Expert Rev Anti Infect Ther. 2015;13:1441–1456. 26536064 10.1586/14787210.2015.1100534

[11] Tissera H Weeraman J Amarasinghe A. Expediency of dengue illness classification: the Sri Lankan perspective. WHO South East Asia J Public Health. 2014;3:5–7. 28607248 10.4103/2224-3151.206884

[12] Morra ME Altibi AMA Iqtadar S. Definitions for warning signs and signs of severe dengue according to the WHO 2009 classification: systematic review of literature. Rev Med Virol. 2018;28:e1979. 29691914 10.1002/rmv.1979

[13] Adam AS Pasaribu S Wijaya H Clinical profile and warning sign finding in children with severe dengue and non-severe dengue. IOP Conf Ser Earth Environ Sci. 2018;125:1–6.

[14] Pothapregada S Sivapurapu V Kamalakannan B. Role of early warning signs in children with severe dengue infection. Int J Contemp Pediatrics. 2018;5:1423.

[15] Tangsathapornpong A Bunjoungmanee P Pengpris P. Comparison of the 1997 and 2009 WHO classifications for determining dengue severity in Thai patients. Southeast Asian J Trop Med Public Health. 2017;48:S75–S82.

[16] Ahmad MH Ibrahim MI Mohamed Z. The sensitivity, specificity and accuracy of warning signs in predicting severe dengue, the severe dengue prevalence and its associated factors. Int J Environ Res Public Health. 2018;15:2018–2012. 30223572 10.3390/ijerph15092018 PMC6163319

[17] Zhang FC Zhao H Li LH. Severe dengue outbreak in Yunnan, China, 2013. Int J Infect Dis. 2014;27:4–6. 25107464 10.1016/j.ijid.2014.03.1392

[18] Leo YS Gan VC Ng EL. Utility of warning signs in guiding admission and predicting severe disease in adult dengue. BMC Infect Dis. 2013;13:498. 24152678 10.1186/1471-2334-13-498 PMC4015176

[19] Wong PF Wong LP AbuBakar S. Diagnosis of severe dengue: challenges, needs and opportunities. J Infect Public Health. 2019;13:193–198. 31405788 10.1016/j.jiph.2019.07.012

