# Supplementary material for: The Value of Warning Signs From the WHO 2009 Dengue Classification in Detecting Severe Dengue in Children
Source: Pediatr Infect Dis J. 2024 Apr 19;43(7):630–4. doi: 10.1097/INF.0000000000004326 (PMC11191040; doi:10.1097/INF.0000000000004326)
Supplement: Supplementary file 1 [file inf-43-630-s001.docx]

Supplemental Digital Content 1. Flow diagram to illustrate the flow of participants through the study.

699 patients with clinical dengue 0-18 years old

509 children

(age 1-14 years)

42 infants

(age< 1 year)

614 confirmed dengue,

evaluated for warning signs

63 adolescents (age >15-18 years)

85 excluded:

65 non-dengue

8 hematology disorder

2 malignancy

10 incomplete data

32

and medical record not complete

Non-severe dengue

26

Severe dengue

16

Non-severe dengue

328

Severe dengue

181

Non-severe dengue

49

Severe dengue

14
