# Supplementary material for: The Value of Warning Signs From the WHO 2009 Dengue Classification in Detecting Severe Dengue in Children
Source: Pediatr Infect Dis J. 2024 Apr 19;43(7):630–4. doi: 10.1097/INF.0000000000004326 (PMC11191040; doi:10.1097/INF.0000000000004326)
Supplement: Supplementary file 2 [file inf-43-630-s002.docx]

**Supplemental Digital Content 1A.** Positive and negative predictive values (PPV/NPV) of combined warning signs in infants

| **Age < 1 year (infants)** | Abdominal pain or tenderness | Persistent vomiting | Clinical fluid accumulation | Mucosal bleeding | Lethargy, restlessness | Liver enlargement > 2 cm | High HCT/low platelets |
| --- | --- | --- | --- | --- | --- | --- | --- |
| Abdominal pain or tenderness | - | 0/61.9 | 0/61.9 | 0/61.9 | 0/61.9 | 0/54.9 | 0/61.9 |
| Persistent vomiting |  | **-** | 0/44.8 | 0/61.9 | 0/61.9 | 0/54.9 | 43.5/66.1 |
| Clinical fluid accumulation |  |  | - | 0/61.9 | 0/54.9 | 67.2/88.0 | 0/61.9 |
| Mucosal bleeding |  |  |  | - | 0/61.9 | 0/61.9 | 0/61.9 |
| Lethargy, restlessness |  |  |  |  | - | 100/76.5 | 0/61.9 |
| Liver enlargement > 2 cm |  |  |  |  |  | - | 0/54.9 |
| High HCT/low platelets |  |  |  |  |  |  | - |

**1B.** Positive and negative predictive values (PPV/NPV) of combined warning signs in children

| **Age 1-14 years (children)** | Abdominal pain or tenderness | Persistent vomiting | Clinical fluid accumulation | Mucosal bleeding | Lethargy, restlessness | Liver enlargement > 2 cm | High HCT/low platelets |
| --- | --- | --- | --- | --- | --- | --- | --- |
| Abdominal pain or tenderness | - | 36.11/64.7 | 66.34/66.9 | 0/63.2 | 0/64.4 | 60.0/66.7 | 60.3/74.8 |
| Persistent vomiting |  | - | 88.9/69.1 | 66.3/66.9 | 0/63.81 | 71.4/70.6 | 53.1/67.9 |
| Clinical fluid accumulation |  |  | - | 0/64.4 | 61.1/65.6 | 88.9/69.1 | 66.3/66.9 |
| Mucosal bleeding |  |  |  | - | 0/64.4 | 51.5/65.3 | 54.8/66.4 |
| Lethargy, restlessness |  |  |  |  | - | 0/64.4 | 0/63.8 |
| Liver enlargement > 2 cm |  |  |  |  |  | - | 73.4/68.5 |
| High HCT/low platelets |  |  |  |  |  |  | - |

**1C.** Positive and negative predictive values (PPV/NPV) of combined warning signs in adolescents

| **Age 15-18 years (adolescents)** | Abdominal pain or tenderness | Persistent vomiting | Clinical fluid accumulation | Mucosal bleeding | Lethargy, restlessness | Liver enlargement > 2 cm | High HCT/low platelets |
| --- | --- | --- | --- | --- | --- | --- | --- |
| Abdominal pain or tenderness | - | 0/66.3 | 0/77.8 | 0/77.8 | 0/77.8 | 0/77.8 | 74.1/100 |
| Persistent vomiting |  | - | 0/77.8 | 0/77.8 | 0/77.8 | 0/77.8 | 0/74.1 |
| Clinical fluid accumulation |  |  | - | 0/77.8 | 100/100 | 0/77.8 | 74.1/100 |
| Mucosal bleeding |  |  |  | - | 0/77.8 | 0/75.9 | 0/77.8 |
| Lethargy, restlessness |  |  |  |  | - | 0/77.8 | 0/77.8 |
| Liver enlargement > 2 cm |  |  |  |  |  | - | 0/77.8 |
| High HCT/low platelets |  |  |  |  |  |  | - |
