# Supplementary material for: The Value of Warning Signs From the WHO 2009 Dengue Classification in Detecting Severe Dengue in Children
Source: Pediatr Infect Dis J. 2024 Apr 19;43(7):630–4. doi: 10.1097/INF.0000000000004326 (PMC11191040; doi:10.1097/INF.0000000000004326)
Supplement: Supplementary file 3 [file inf-43-630-s003.docx]

**Supplemental Digital Content 3**. Performance of combinations of two or more Warning Signs in relation to detecting severe dengue on admission in different age groups.

| **Warning Signs** | **PPV** | **NPV** | **Sn** | **Sp** |
| --- | --- | --- | --- | --- |
| **Age < 1 year (infants)** |  |  |  |  |
| Two WSs | 37.5% | 61.5% | 37.5% | 61.5% |
| Three WSs | 66.7% | 66.7% | 25.0% | 92.3% |
| Four WSs | 100% | 63.4% | 6.3% | 100% |
|  |  |  |  |  |
| **Age 1-14 years (children)** |  |  |  |  |
| Two WSs | 31.9% | 63.0% | 24.9% | 70.7% |
| Three WSs | 52.2% | 68.0% | 25.9% | 86.9% |
| Four WSs | 62.5% | 68.3% | 22.1% | 92.7% |
|  |  |  |  |  |
| **Age 15-18 years (adolescents)** |  |  |  |  |
| Two WSs | 23.5% | 78.3% | 28.6% | 73.5% |
| Three WSs | 33.3% | 80.4% | 28.6% | 83.7% |
| Four WSs | 50.0% | 79.7% | 14.3% | 95.9% |
